# Chrononutrition and Physical Fitness in Schoolgirls Aged 10–14 Years: Associations with Obesity Risk

**DOI:** 10.3390/nu18091441

**Published:** 2026-04-30

**Authors:** Hessa A. Alhabib, Shaea A. Alkahtani, Maha H. Alhussain

**Affiliations:** 1 Department of Food Sciences and Nutrition, College of Food and Agricultural Sciences, King Saud University, P.O. Box 2460, Riyadh 11451, Saudi Arabia; 443203530@student.ksu.edu.sa; 2 Exercise Physiology Department, College of Sport Sciences and Physical Activity, King Saud University, P.O. Box 2460, Riyadh 11451, Saudi Arabia; shalkahtani@ksu.edu.sa

**Keywords:** chrononutrition, obesity, physical fitness, VO_2_ max, female, school student, Saudi Arabia

## Abstract

Chrononutrition, which emphasizes the timing, frequency, and regularity of eating in alignment with circadian rhythms, has emerged as an important yet understudied determinant of obesity, particularly in children and adolescents. We aimed to compare chrononutrition and physical fitness between elementary and intermediate schoolgirls and to examine their associations with obesity. **Methods:** This cross-sectional study included 457 schoolgirls aged 10–14 years from elementary and intermediate schools. Chrononutrition behaviors were evaluated. Anthropometric measurements and physical fitness, including handgrip strength, standing long jump, and 20 m shuttle run, were assessed. Fasting blood glucose and lipid profile were determined using capillary blood samples. **Results:** Compared with intermediate students, elementary school students demonstrated more favorable meal-related behaviors, longer overnight fasting durations, and better dietary intake (*p* < 0.05), along with higher VO_2_ max and higher standing long jump performance. Conversely, intermediate students exhibited greater absolute handgrip strength. A higher number of meals/day (aOR = 0.68, *p* = 0.039) and a longer interval between the last meal and bedtime (aOR = 0.78, *p* = 0.013) were inversely associated with obesity. Furthermore, higher HGS/BMI was associated with lower odds of obesity (aOR = 0.01, *p* < 0.001), while HDL cholesterol was inversely associated with obesity (aOR = 0.91, *p* < 0.001). **Conclusions:** Chrononutrition behaviors and physical fitness varied across school stages and were associated with obesity among school-aged girls. Higher meal frequency, a longer interval between the last meal and bedtime, and greater handgrip strength relative to body mass index (HGS/BMI) were associated with lower odds of obesity. Non-obese students also demonstrated higher VO_2_ max. These findings suggest that chrononutrition behaviors and physical fitness may contribute to obesity prevention, underscoring the importance of early nutrition and physical activity interventions.

## 1. Introduction

In recent years, increasing attention has been directed toward chrononutrition, emphasizing that when and how consistently meals are eaten during the day are as important as what people eat in shaping overall health [1]. Chrononutrition is the study of the interaction between circadian rhythms and nutrition, focusing on how the timing of food intake, synchronized with the body’s biological clock, influences metabolic processes and, ultimately, human health [2]. It integrates concepts from chrono-biology and nutrition to examine how the timing of eating affects health consequences [3]. This concept encompasses three primary dimensions of eating behavior: (1) timing (the specific time of day when meals are consumed), (2) frequency (number of meals consumed daily), and (3) regularity (variability in energy intake across meals throughout the day or from day to day) of food intake [4].

Research on both humans and animals has shown that the timing of eating plays a critical role in health [5]. Eating at a later time has been linked to increased fat accumulation and disruptions in glucose regulation [6]. Present theories suggest a link between meal timing and body weight related to the physiological effects of disrupted circadian rhythms in sleep and eating patterns [4]. Chrononutrition has also been linked to metabolic biomarkers [7], cardiovascular disease, type 2 diabetes, hypertension, and obesity [8].

Despite growing interest in chrononutrition as a promising approach to improve human health, existing research has focused on adults, leaving a critical gap in children and adolescents, who differ from adults in both physiological and psychological characteristics. Adolescents are especially vulnerable to circadian dysfunction because discrepancies between their sleep–wake patterns and social schedules often result in delayed bedtimes and wake times. Studies have shown that circadian misalignment in adolescents is associated with an increased risk of obesity [9]. Nonetheless, there is a scarcity of population-based studies assessing chrononutrition behaviors in pediatric groups [8,10]. Nutrient requirements, dietary habits, and social life around eating undergo significant changes as children age. Younger children usually snack at home in the morning, whereas school-age children and adolescents tend to snack later in the day and more often outside the home [11]. Weight status in this age group is shaped by a complex interplay of factors within the home, school, and broader community environment [12]. Parental feeding practices and household routines may influence daily eating patterns [13,14], while school schedules may also shape the regularity and timing of meals [15]. Examining the role of chrononutrition in children and adolescents is crucial for understanding its potential effects on metabolic health outcomes.

In addition to chrononutrition, physical fitness is recognized as a key determinant of overall health. Physical fitness is considered a multi-component construct and an important factor of health in children and adolescents [16]. Low physical fitness, particularly reduced muscular strength and cardiorespiratory fitness, has been associated with increased adiposity and a higher risk of obesity among children and adolescents [17,18,19]. Individuals with higher levels of physical fitness generally demonstrate lower body fat and more favorable metabolic profiles, independent of body mass index (BMI) [17,18,20]. Evaluating physical fitness alongside other lifestyle behaviors provides a more comprehensive understanding of obesity risk in childhood and adolescence.

Childhood obesity is a public health concern in Saudi Arabia [21,22]. It has been reported that overweight and obesity prevalence among Saudi children aged 5–16 years in the Qassim region, one of the regions of Saudi Arabia, was 13.6% and 23.9%, respectively [23]. In this context, a study conducted in the region examined the dietary behaviors of adolescents and reported concerning patterns, with only 27.7% consuming breakfast daily [24]. Comparable data on dietary behaviors and physical activity among school-aged children in the Qassim region of Saudi Arabia are limited. Understanding the role of chrononutrition and physical fitness in obesity may help inform public health strategies and support the development of a low-cost, effective approach to preventing obesity and promoting healthier habits during critical developmental years. Therefore, the aim of this study was to compare chrononutrition and physical fitness between elementary and intermediate schoolgirls aged 10–14 years and to examine their associations with obesity. We hypothesized that obesity among school-aged girls would be associated with unfavorable chrononutrition patterns, reduced physical fitness, and adverse biochemical markers.

## 2. Materials and Methods

### 2.1. Participants

This cross-sectional study was conducted in Buraidah city, Al-Qassim region, Saudi Arabia, from December 2024 to April 2025. Female students were recruited from elementary and intermediate schools, using a multistage stratified cluster sampling design based on geographic areas within the city and school type (private vs. public). Twelve schools (six elementary and six intermediate) were randomly selected from the three main educational branch offices in Buraidah city (East, West, and Central).

All the students aged 10 to 14 years enrolled in the girls’ elementary and intermediate schools during the study period were eligible for inclusion in the study. Those with physical impairments, medical conditions that interfere with the normal eating habits or participation in physical activity (e.g., eating disorders, cardiovascular disease, type 1 diabetes mellitus), or use of melatonin or other medications that affect sleep were excluded.

**Figure 1:**
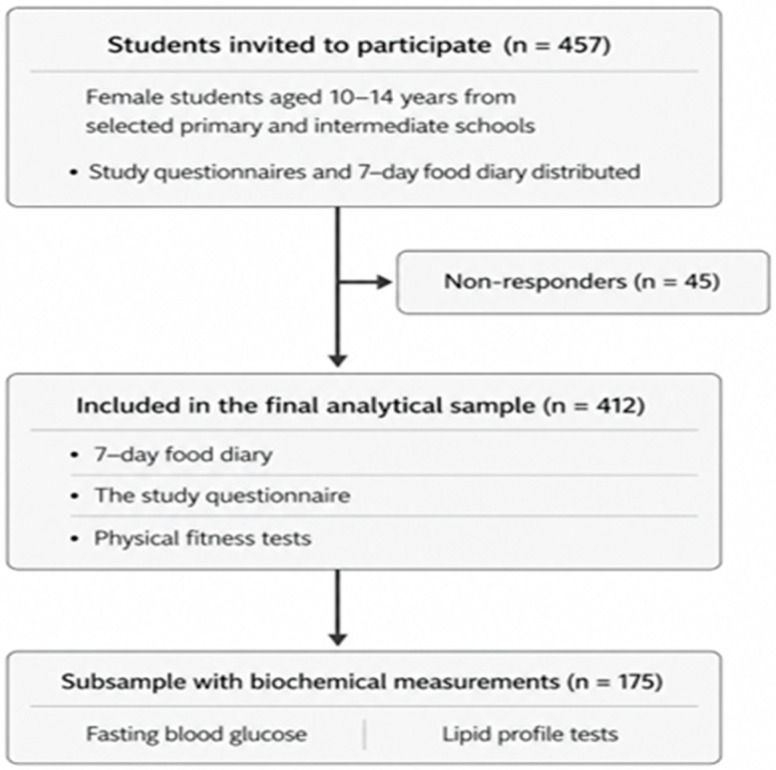
Participant recruitment and enrollment flowchart.

Permission to conduct the study in the selected schools was granted by the Ministry of Education. Official letters were sent to the schools to request their collaboration and facilitate implementation of the study procedure. Invitation letters were delivered to approximately 100–150 parents per school. In total, 457 parents provided affirmative responses, with an average of approximately 40 responses per school and a minimum of 12 responses in one school. Of the 457 students invited to participate, 45 were excluded from the study due to failure to provide a food diary or complete the study questionnaire (Figure 1).

This study was approved by the institutional review board of King Saud University (IRB: KSU-HE-24-682). Written informed consent was obtained from all parents/guardians. The study procedures were explained to the students in an age-appropriate manner, and verbal assent was obtained before participation. Students and their parents were informed that participation was voluntary and that they could withdraw at any time if they felt uncomfortable.

The required sample size for this cross-sectional school-based study was estimated, assuming a population proportion that yields the maximum possible sample size (*p* = 0.50), with a 95% confidence level and a 5% margin of error. To account for the clustered sampling design, potential non-responses, and incomplete data, the sample size increased by 15%, resulting in a final minimum sample of 404 students.

### 2.2. Study Measurements

On school days, all assessments were conducted in the morning between 7:30 and 11:00 by a trained researcher in accordance with standardized protocols. Anthropometric measurements, body composition and handgrip strength were assessed. Physical fitness assessments including the 20 m shuttle run and the standing long jump were also assessed and were carried out on the school’s field. During the 20 m shuttle run, at least one researcher or research assistant supervised every ten students to ensure proper test administration and safety.

Anthropometry measures: Height was taken to the nearest 0.1 cm using a calibrated measuring rod, while the student was standing barefoot. Body weight and composition were assessed through the bioelectrical impedance analysis (BIA) method using a single-frequency, tetrapolar, hand-to-foot electronic scale (Omron BF-511, Omron Healthcare Co., Kyoto, Japan). Prior to measurements, the previously recorded height, age, and sex of the students were entered into the device using its keypad. Students were measured without shoes and in light clothes, in accordance with the manufacturer’s guidelines.

BMI was calculated by dividing weight in kilograms by height in meters squared (kg/m^2^). BMI cut-off reference standards by age and sex from the International Obesity Task Force (IOTF) were employed to classify thinness, normal weight, overweight, and obesity according to the child’s age and sex [25].

Dietary intake assessment: Dietary intake was evaluated using a 7-day food diary record, in which parents/guardians were asked to document all foods and beverages consumed by their children in real time during the recording period to minimize recall bias. To improve reporting accuracy, detailed instructions were provided, and standardized portion size guides were used. The diaries were reviewed by a trained dietitian to verify entries and clarify any ambiguities. Implausible energy intakes were identified based on expected intake ranges for age and sex and were reviewed before inclusion in the final analysis. Food diaries were considered valid if they included at least two weekdays and one weekend day.

Dietary data were analyzed using Food Processor software version 16 (ESHA Research, Inc., Salem, OR, USA) to estimate daily energy and macronutrient intake. A day was defined analytically as starting when the student woke up for school and ending when they went to bed at night. Caloric intake was categorized into two time-based groups: energy consumed before 6:00 P.M. (morning energy intake) and energy consumed at or after 6:00 P.M. (evening energy intake). Consistent with previous pediatric studies categorizing afternoon/after-school (3:00–6:00 P.M.) and evening (6:00–9:00 P.M.) periods, a 6:00 P.M. cut-off was applied [11]. The percentage of daily caloric intake consumed before 6:00 P.M. was computed as follows:

$$100\;\times \;\frac{Calories\;before\;6\;P.M.}{Total\;daily\;calories}=Calories\;before\;6\;P.M.\%$$

Similarly, the percentage of calories consumed after 6:00 P.M. was calculated in relation to total daily consumption.

Chrononutrition assessment: Chrononutrition variables were derived from both a structured questionnaire and the 7-day food diary, which provided information on number of meals consumed, meal timing and frequencies, and overnight fasting. The number of meals was defined according to a minimum intake of ≥50 kcal separated by at least 15 min, consistent with previous chrononutrition research [26]. Fasting duration was calculated as the time interval, in hours, between the last eating episode of one day and the first eating episode of the subsequent day, subtracted from 24 h as the method described by [27]. Breakfast timing was categorized relative to the start of the school day, as follows: early breakfast (consumed before 7:15 A.M.) and a late breakfast (consumed after 7:15 A.M.), given that the school day commenced at 7:30 A.M. Lunchtime was also categorized into early lunch (before 1:00 P.M.) and late lunch (after 1:00 P.M.), depending on the school day’s end time. Dinner times were categorized based on a cut-off time of 7:30 P.M., which is typical of sleep schedules for school-aged students. Students who eat dinner before 7:30 P.M. are considered early, and those who eat dinner after that time are considered late.

Biochemical measurements: Fasting blood glucose (FBG) and lipid profile, including total cholesterol, Low-Density Lipoprotein (LDL), High-Density Lipoprotein (HDL), and triglycerides, were determined from capillary blood samples obtained via finger-stick, after a 12 h overnight fast. Samples were collected from the students by trained staff. Biochemical analyses were performed using a Cardio Check^®^ Plus Analyzer (CCPA) (PTS Diagnostics, Indianapolis, IN, USA). The reliability and validity of CCPA have been reported for the analysis of blood glucose and lipids [28]. Students rested for at least 10 min, after which they took their turns on the test. Each student’s middle finger was cleaned with an alcohol swab, after which gentle pressure was applied with a lancet, and the finger was pricked at its center. Meanwhile, gentle pressure was applied to the finger to produce a large drop of blood, which was dispensed on the test strip, and test results were displayed on the analyzer within 90 s.

### 2.3. Physical Fitness

Handgrip strength assessment: An isometric handgrip dynamometer (Baseline^®^ Smedley spring-type dynamometer, Fabrication Enterprises, Inc., White Plains, NY, USA) was used to evaluate muscle strength. Using the dominant hand, students get a handle on the dynamometer and apply a difficult weight. Each student made two endeavors, and the most elevated attempt’s perusing was recorded in kg. Amid the execution, the arm holding the dynamometer must not swing and must not come into contact with the student’s body or any exterior objects. Relative handgrip strength (HGS/BMI) was also calculated by dividing handgrip strength (kg) by BMI (BMI, kg/m^2^).

The 20 m shuttle run: A modified version of the 20 m shuttle run test, known as the FITNESSGRAM PACER [29], measures cardiorespiratory fitness. Conducted on a 20 m course marked by cones, students run back and forth in time with an audio cue, with the speed increasing by 0.5 km/h per minute from an initial speed of 8.5 km/h. According to [30], the test ends when students reach voluntary fatigue or fail to maintain the necessary speed twice. Performance was quantified by the total shuttles completed and the estimated maximal oxygen uptake (VO_2_ max, mL·kg^−1^·min^−1^).

Standing long jump: the strength of the lower body limbs was examined by stepping on a line and pushing forward, with the distance measured from their heel to the ground. The highest score from two attempts was recorded in centimeters [31].

Statistical Methods: The obtained data were cleaned, organized, coded, and analyzed using the Statistical Package of Social Science V.26 (SPSS Inc., Chicago, IL, USA). Descriptive statistics were presented as mean ± standard deviation (SD) for continuous variables and as frequencies and percentages (%) for categorical variables. Kolmogorov–Smirnov test was used to check the normality for all continuous variables. Categorical variables were tested using the Chi-square test. Continuous variables were tested using the independent *t*-test. Binary logistic regression analysis was performed to examine the associations between study variables and obesity status. Statistical significance was considered when *p*-values were less than 0.05.

## 3. Results

A total of 412 students (49.8% from elementary schools and 50.2% from intermediate schools) were included in this study. The response rate by parents/guardians was 90.2%. The majority of the questionnaires were completed by mothers (76.5%), followed by fathers (18.4%), while a small proportion was completed by guardians (5.1%). Descriptive characteristics of the students, including body composition measures derived from bioimpedance analysis, are displayed in Table 1. The mean age was 11.82 ± 1.28 years, with a range of 10 to14 years. According to BMI-for-age classification, the majority of participants had normal weight (36.9%), followed by overweight (26.0%), obesity (23.5%) and thinness (13.6%).

Chrononutrition variables, for the total sample and stratified by school stage, are shown in Table 2. Comparisons between the elementary and intermediate students were performed. Significant differences between elementary and intermediate school students were observed in several chrononutrition variables, including eating the same number of meals daily, breakfast time/day, lunch and dinner regularity, fasting hours/night, total daily energy intake (kcal/day), morning energy intake (before 6 P.M. (kcal)), total fiber (g/day), serving fruit eaten/day, fruit consumed/cup/day (*p* < 0.05).

Regarding whether the student eats the same number of meals daily, the response “yes” was more commonly reported among elementary school students (62.6%) compared with intermediate school students (37.4%), whereas the response “no” answer was more frequently reported among intermediate school students (58.0%) than elementary school students (42.0%). Early breakfast was more common among the intermediate students (57.6%) than the elementary students (42.4%). On the other hand, late breakfast was reported more frequently among the elementary students (54.6%) than the intermediate students (45.4%).

Similar patterns were observed for both lunch and dinner consumption. Regular meal consumption was more common among elementary students than intermediate students for lunch (54.3% vs. 45.7%) and dinner (54.7% vs. 45.3%). Conversely, irregular consumption was more frequently reported among intermediate students than elementary students for lunch (64.4% vs. 35.6%) and dinner (62.9% vs. 37.1%). In terms of fasting hours/night, a longer fasting period was observed among elementary school students compared with intermediate school students (11:20 ± 2:10 vs. 10:28 ± 2:36 h/night), with a statistically significant difference between the two groups (*p* < 0.001).

Regarding dietary intake, elementary school students reported higher values than intermediate school students for both total daily energy intake (1611.93 ± 366.88 vs. 1488.97 ± 409.73 kcal/day) and morning energy intake before 6 P.M. (1053.61 ± 284.70 vs. 960.40 ± 300.53 kcal), with statistically significant differences between the groups (*p* = 0.001). Elementary school students reported higher fiber intake than intermediate school students (10.99 ± 4.15 vs. 9.94 ± 4.59 g/day). Similarly, fruit consumption was higher among elementary students than intermediate students in terms of both daily servings (0.37 ± 0.41 vs. 0.25 ± 0.28 servings/day) and cups per day (0.18 ± 0.20 vs. 0.12 ± 0.14 cups/day), with statistically significant differences between the groups (*p* ≤ 0.015).

On the other hand, no significant differences were found between the two groups regarding the number of meals per day, number of meals/days, breakfast regularly, lunch and dinner time/day, time between last meal and bedtime hours/night, most meals are important for the child, eat snacks between meals, evening energy intake (after 6 P.M. (kcal)), serving vegetables eaten/day, vegetables consumed/cup/day (*p* > 0.05).

Table 3 shows physical fitness according to school stage. VO_2_ max (mL/kg/min) was significantly higher among elementary students than intermediate students (*p* = 0.008), indicating better cardiorespiratory fitness in the elementary group. Intermediate students demonstrated significantly greater handgrip strength and HGS/BMI than elementary students (*p* < 0.001). In addition, elementary students achieved significantly longer standing long jump distances than intermediate students (*p* = 0.006).

Table 4 demonstrated chrononutrition variables and dietary intake based on BMI categories. Significant differences between non-obese and obese students were observed in chrononutrition variables, including number of meals/day, breakfast time/day, total daily energy intake (kcal/day), morning energy intake (before 6 P.M. (kcal)), evening energy intake (after 6 P.M. (kcal)) (*p* < 0.05).

As shown in the table, non-obese students reported a significantly higher mean number of daily meals compared with obese students (*p* < 0.001). There was also a significant association between the two groups and breakfast time (*p* = 0.008). A greater proportion of non-obese students ate an early breakfast (55.4%), whereas obese students were more likely to eat late breakfast (58.2%). Total daily energy intake was significantly higher among non-obese students compared with obese students (*p* = 0.002). Morning energy intake (morning to 6 P.M.) and evening energy intake (after 6 P.M.) were significantly higher among non-obese students compared with obese students (*p* = 0.017, 0.050).

There were no significant differences between the two groups for the other chrononutrition and dietary variables regarding eating the same number of meals daily, breakfast regularly, lunch and dinner time/day, lunch and dinner regularity, fasting hours/night, time between last meal and bedtime hours/night, most meals are important for the child, eat snacks between meals, total fiber (g/day), serving vegetables eaten/day, vegetables consumed/cup/day, serving fruit eaten/day, fruit consumed/cup/day (*p* > 0.05).

As shown in Table 5, non-obese students had significantly higher VO_2_ max (mL/kg/min) than obese students (*p* < 0.001), indicating better cardiorespiratory fitness in the non-obese group. In contrast, obese students demonstrated significantly higher handgrip strength than their non-obese counterparts (*p* < 0.001). On the other hand, non-obese students had significantly higher HGS/BMI than obese students (0.84 ± 0.21 vs. 0.68 ± 0.18, *p* < 0.001).

FBG and lipid profile levels for the total sample and by BMI categories (non-obese and obese) are shown in Table 6. HDL was the only variable with a statistically significant difference between the two groups (*p* ≤ 0.001), with a higher mean in non-obese students than in obese students. In contrast, FBG and the other lipid profile did not differ significantly between the two groups.

Binary logistic regression analysis of chrononutrition, physical fitness, and biochemical variables in relation to obesity status is presented in Table 7. The notable associations identified in the unadjusted models persisted as statistically significant following age adjustment. As shown in the table, a higher number of meals/days was significantly associated with lower odds of obesity (aOR = 0.68, 95% CI: 0.46–0.98, *p* = 0.039). In addition, a prolonged time between the last meal and bedtime hours/night was inversely correlated with obesity (aOR = 0.78, 95% CI: 0.64–0.95, *p* = 0.013). Higher HGS/BMI was significantly associated with lower odds of obesity (aOR = 0.01, 95% CI: 0.002–0.030, *p* < 0.001). HDL was inversely associated with obesity (aOR = 0.91, 95% CI: 0.87–0.96, *p* < 0.001).

## 4. Discussion

The present study explored the associations of chrononutrition behaviors and physical fitness including 20 m shuttle run, handgrip strength, and standing long jump with obesity among Saudi girls aged 10–14 years. The study findings showed that chrononutrition behaviors change with age, with significant variations observed in certain patterns between elementary and intermediate school students. Differences were observed in the number of meals per day, breakfast time, lunch and dinner regularity, fasting duration, total daily energy intake, morning energy intake fiber, and fruit consumption. Developmental and lifestyle changes associated with school stages, such as increased autonomy over food choices and altered daily routines, may affect chrononutrition and dietary intake patterns in children. As children transition from childhood into early adolescence, variability in eating habits can occur. Age-related changes in eating habits can lead to less structured dietary patterns as they gain independence in food choices and routines [32]. It has been reported that eating habits often change from childhood to adolescence, frequently accompanied by a decline in diet quality and greater variability in the patterns [33]. Understanding these stage-specific variations in chrononutrition behaviors may assist in formulating age-appropriate nutritional strategies to foster healthy eating habits among school-aged children.

With regards to physical fitness, the current study also showed notable disparities in physical fitness indices across elementary and intermediate school students. Elementary school students demonstrated higher values in VO_2_ max. The 20 m shuttle run test, a widely used measure of cardiorespiratory fitness in youth, may be influenced by physical activity levels, growth patterns, and body composition. Handgrip strength was stronger in intermediate schoolgirls, which can be attributed to pubertal changes associated with increases in muscle mass and strength. Handgrip strength progressively increases with age throughout childhood and adolescence, as part of normal growth and musculoskeletal development [34]. Similarly, when handgrip strength was expressed relative to BMI, intermediate school students still showed significantly higher values than elementary school students. This suggests that the greater handgrip strength observed in intermediate students may not be explained solely by differences in body size but may also reflect developmental and maturational differences in muscular fitness across school stages. Elementary school students demonstrated higher performance in the standing long jump compared with intermediate school students. While muscle strength often escalates with age, performance in explosive lower limb tasks does not always improve during early adolescence. Prior research indicated that girls’ jumping performance may be either maintained or diminished during this growing period, potentially associated with changes in body mass [20]. These changes are linked to increased body mass, which is typical at this stage of development. As children gain weight, their strength relative to their body size decreases, making it harder for their legs to push off the ground. It has been shown that children with higher weight for height are likely to exhibit poorer performance in movements, such as the standing broad jump, compared with their normal-weight peers [35].

Regarding chrononutrition in relation to obesity, non-obese students reported consuming more meals per day on average than obese students. Logistic regression also supported this, showing that an increased number of meals per day is associated with a reduced likelihood of obesity. These findings suggest that eating more meals throughout the day may be related to more regular eating habits in children and adolescents. It has been reported that meal frequency and timing are associated with the risk of being overweight and obese [10,36]. A recent study showed that children and adolescents who eat four or more meals a day may have a lower risk of overweight or obesity than those who eat three or fewer meals a day [10]. Furthermore, a longitudinal study indicated that a higher meal frequency (five meals per day versus three or fewer meals per day) was linked to a reduced increase in BMI-z score, a diminished increase in waist-to-height ratio, and lower odds of developing obesity at follow-up [37]. It has been suggested that a higher frequency of meals or snacks throughout the day is associated with a lower risk of being overweight and obese in children, particularly when eating patterns are regular and total energy intake does not increase [38]. Eating more meals throughout the day may help children and adolescents maintain a healthy weight and energy balance by reducing the chance of overeating at a single meal and spreading out their calories more evenly. However, these results should be interpreted with caution as they might be influenced by additional variables, including total energy intake, diet quality, and physical activity level. Furthermore, the total daily energy intake findings in the present study warrant careful interpretation. Previous evidence indicates that misreporting of energy intake is common in children and adolescents [39] and that adolescents with overweight or obesity may be more likely to underreport their energy intake than their normal-weight peers [40]. In this context, an unexpected finding in the present study was that non-obese students reported higher total daily energy intake than obese students. This apparently paradoxical result may, therefore, partly reflect greater underreporting among participants with obesity rather than a true lower energy intake. The present study found that the time interval between the last meal and bedtime is a significant indicator of chrononutrition associated with obesity. A longer interval between the last meal and bedtime was associated with a lower risk of obesity. These results suggest that the timing of the last meal can independently predict obesity risk, and this effect remains significant after accounting for age. The group analysis revealed a consistent pattern: obese students exhibited a shorter average interval between their last meal and bedtime compared with non-obese students. However, this difference did not reach statistical significance. This may be attributed to the lower statistical power of simple comparative tests compared with regression models, which more effectively account for relationships among variables, such as obesity. Taken together, these findings support the growing evidence that the temporal distribution of food intake, particularly the proximity of eating to sleep, may represent an important behavioral component of chrononutrition linked to obesity risk. These results can be explained in light of the circadian rhythm of metabolic processes in the human body. Evidence suggests that the timing of food intake relative to the sleep–wake cycle plays a significant role in regulating glucose and lipid metabolism, as human metabolism exhibits a clear circadian rhythm that makes the body’s response to food more efficient during daylight hours compared to evening hours [41,42]. As evening progresses, melatonin levels rise. This hormone is associated with decreased insulin sensitivity and a reduced ability of pancreatic cells to produce insulin. This leads to impaired glucose tolerance when eating late at night. Consequently, eating close to bedtime may coincide with a period of decreased metabolic efficiency, resulting in elevated post-meal glucose levels and increased fat storage in the long term [41]. These results indicate that a longer time interval between the last meal and bedtime is associated with a lower likelihood of obesity, suggesting that it may help align energy intake timing with biological metabolic rhythms.

Recent studies showed that eating close to bedtime disrupts meal timing and the body’s circadian rhythm, leading to circadian misalignment. This condition is associated with impaired glucose and insulin regulation, changes in appetite hormones, and energy balance disturbances, which heighten the risk of obesity and metabolic disorders in adolescents and children [10,43]. Consistent with this evidence, previous research has shown that the proximity of the last meal to sleep may influence indicators of adiposity in children. A study involving children found that eating their final meal close to bedtime was associated with an elevated waist-to-height ratio, a significant marker of central obesity. These indicate that the timing of meals relative to sleep may significantly impact children’s metabolic health [44].

In addition to biological factors, the relationship between meal timing and obesity may be influenced by lifestyle behaviors. Individuals who eat closer to bedtime often follow later bedtimes or more irregular daily routines, patterns that are frequently accompanied by shorter sleep duration or lower levels of physical activity. Together, these behaviors may create metabolic and behavioral conditions that promote positive energy balance and weight gain. The findings of this study indicated that the interval between the last meal and bedtime may serve as a significant behavioral marker of temporal chrononutrition linked to obesity risk. Furthermore, these results align with recent trends in nutrition research that emphasize the importance of energy timing in regulating energy balance and metabolic health, alongside the traditional focus on food quantity and quality.

Regarding physical fitness, the present study showed that obese students had greater handgrip strength compared with their non-obese counterparts, whereas non-obese students had significantly higher HGS/BMI. Higher HGS/BMI was significantly associated with lower odds of obesity in both crude and age-adjusted logistic regression models. Although muscular strength is typically considered a favorable health indicator, our findings underscore the difference between absolute and relative strength. Absolute grip strength reflects total muscle force and may increase with body size and muscle mass, whereas relative strength, which accounts for body size, may better reflect muscular fitness. Consequently, the elevated handgrip strength reported among obese students in this study may indicate the impact of increased body size rather than higher muscular fitness. Previous research has also suggested that interpreting handgrip strength in children should consider body size, as weight-specific grip strength may be a more accurate indicator of muscular fitness and cardiometabolic risk [45].

In the present study, HDL levels were significantly lower among obese students compared with their non-obese peers, and higher HDL levels were associated with lower odds of obesity in the logistic regression analysis. This inverse relationship is consistent with previous evidence showing that childhood obesity is frequently accompanied by adverse lipid profiles, particularly reduced HDL levels [46]. Clustering of cardiovascular disease risk factors, including elevated LDL, increased triglycerides, and reduced HDL levels, has been strongly associated with obesity in children [47]. It has been shown that decreased HDL is one of the most common lipid abnormalities among overweight and obese children and adolescents [48].

A key strength of this study is its comprehensive assessment of chrononutrition behaviors among youth girls, an emerging area of research. Chrononutrition was assessed using both a structured questionnaire and the food records, allowing for a more detailed and reliable assessment of meal patterns. Nevertheless, this study has several limitations that should be acknowledged. Given the cross-sectional design, we cannot determine cause-and-effect relationships. The sample included only female students from one region, which may limit the generalizability of the study findings. Furthermore, underreporting of daily energy intake is a common and acknowledged source of measurement error in the assessment of food intake.

## 5. Conclusions

In conclusion, significant differences in chrononutrition behaviors and physical fitness were observed between elementary and intermediate schoolgirls, with specific patterns associated with obesity risk. More meals per day, a longer interval between the last meal and bedtime, and higher HGS/BMI were associated with lower odds of obesity. These findings highlight the potential importance of meal timing, meal frequency, and physical fitness in early obesity prevention. Integrating chrononutrition principles into pediatric health strategies may enhance efforts to reduce obesity in this population. From a practical perspective, school-based nutrition policies may benefit from promoting more regular meal patterns, supporting healthier timing of food intake, and encouraging healthy lifestyle behaviors. Further longitudinal studies are required to clarify the potential influence of feeding timing on obesity development.

## Figures and Tables

**Table 1:** Descriptive characteristics of the study sample.

Variables	ALL (*n* = 412)
Age (years)	11.82 ± 1.28
Height (cm)	144.98 ± 8.80
Weight (kg)	46.06 ± 13.13
BMI (kg/m^2^)	21.70 ± 4.92
Body Fat (%)	26.91 ± 11.26
Muscle mass (kg)	30.41 ± 3.60
BMI IOTF	
Thinness	56 (13.6)
Normal	152 (36.9)
Overweight	107 (26.0)
Obese	97 (23.5)

Data are presented as means ± SD or frequencies (%). BMI: body mass index; IOTF: International Obesity Task Force.

**Table 2:** Chrononutrition variables of the study sample according to the school stage.

Variables	ALL (*n* = 412)	Elementary (*n* = 205)	Intermediate (*n* = 207)	*p*-Value
**Age (years)**	11.82 ± 1.28	10.77 ± 0.74	12.86 ± 0.74	<0.001 *
**Number of meals/days**	4.09 ± 0.68	4.03 ± 0.68	4.14 ± 0.69	0.103
**Eat the same number of meals daily** Yes No	155 (37.5%) 257 (62.5%)	97 (62.6%) 108 (42.0%)	58 (37.4%) 149 (58.0%)	<0.001 *
**Breakfast Time/day** Early breakfast (Before School) Late breakfast (in the school)	158 (38.3) 251 (60.9)	67 (42.4%) 137 (54.6%)	91 (57.6%) 114 (45.4%)	0.016 *
**Breakfast regularly** Yes No	261 (63.4%) 151 (36.6%)	137 (52.5%) 68 (45.0%)	124 (47.5%) 83 (55.0%)	0.135
**Lunch Time/day** Early Lunch (before 1 P.M.) Late Lunch (after 1 P.M.)	75 (18.2) 336 (81.6)	32 (42.7%) 172 (51.2%)	43 (57.3%) 164 (48.8%)	0.182
**Lunch regularly** Yes No	311 (75.5%) 101 (24.5%)	169 (54.3%) 36 (35.6%)	142 (45.7%) 65 (64.4%)	0.001 *
**Dinner Time/day** Early Dinner (before 7:30 P.M.) Late Dinner (after 7:30 P.M.)	37 (9.0) 372 (90.3)	23 (62.2%) 182 (48.9%)	14 (37.8%) 190 (51.1%)	0.125
**Dinner regularly** Yes No	296 (71.7%) 116 (28.3%)	162 (54.7%) 43 (37.1%)	134 (45.3%) 73 (62.9%)	0.002 *
**Fasting Hours/night**	10:54 ± 2:26	11:20 ± 2:10	10:28 ± 2:36	<0.001 *
**Time between last meal and bedtime hours/night**	1:50 ± 1:10	1:45 ± 1:02	1:55 ± 1:17	0.151
**Most meals are important for the child** Breakfast Lunch Dinner	176 (42.6%) 182 (44.3%) 54 (13.1%)	90 (51.1%) 91 (50.5%) 24 (44.4%)	86 (48.9%) 91 (49.5%) 30 (55.6%)	0.684
**Eat snacks between meals** Yes No	366 (88.9%) 46 (11.1%)	180 (49.2%) 25 (54.3%)	186 (50.8%) 21 (45.7%)	0.520
**Total daily energy intake (kcal/day)**	1550.15 ± 393.37	1611.93 ± 366.88	1488.97 ± 409.73	0.001 *
**Morning energy intake—before 6 P.M. (kcal)**	1006.66 ± 296.12	1053.61 ± 284.70	960.40 ± 300.53	0.001 *
**Evening energy intake—after 6 P.M. (kcal)**	547.48 ± 210.56	567.1837 ± 197.89	528.06 ± 221.10	0.060
**Total fiber (g/day)**	10.46 ± 4.40	10.99 ± 4.15	9.94 ± 4.59	0.015 *
**Serving vegetables eaten/day**	0.26 ± 0.27	0.27 ± 0.29	0.26 ± 0.24	0.665
**Vegetable consumed/cup/day**	0.13 ± 0.13	0.13 ± 0.14	0.13 ± 0.12	0.721
**Serving fruit eaten/day**	0.31 ± 0.35	0.37 ± 0.41	0.25 ± 0.28	<0.001 *
**Fruit consumed/cup/day**	0.15 ± 0.18	0.18 ± 0.20	0.12 ± 0.14	<0.001 *

Data are presented as means ± SD or frequencies (%). Categorical variables were tested using the Chi-square test and continuous variables were tested using the independent *t*-test. * *p* < 0.05 was considered statistically significant.

**Table 3:** Physical fitness of the study sample according to the school stage.

Variables	ALL *n* = 412	Elementary *n* = 205	Intermediate *n* = 207	*p*-Value
**20 mSRT**				
Total Laps	10.39 ± 9.14	10.67 ± 11.59	10.12 ± 5.83	0.544
VO_2_ MAX = mL/kg/min	32.97 ± 4.11	33.52 ± 4.14	32.45 ± 4.03	0.008 *
**HGS (kg)**	16.10 ± 4.46	14.55 ± 4.07	17.61 ± 4.32	<0.001 *
**HGS/BMI**	0.76 ± 0.21	0.72 ± 0.21	0.80 ± 0.20	<0.001 *
**SLJ (cm)**	132.48 ± 31.10	136.74 ± 29.18	128.27 ± 32.42	0.006 *

Data are presented as means ± SD or frequencies (%). Categorical variables were tested using the Chi-square test and continuous variables were tested using the independent *t*-test. * *p* < 0.05 was considered statistically significant; 20 mSRT: 20 m shuttle run test; HGS: handgrip strength; HGS/BMI: relative handgrip strength/body mass index; SLJ: standing long jump.

**Table 4:** Chrononutrition variables of the study sample according to BMI categories.

Variable	ALL (*n* = 412)	Non-Obese (*n* = 208)	Obese (*n* = 204)	*p*-Value
**Number of meals/days**	4.09 ± 0.68	4.21 ± 0.67	3.96 ± 0.68	<0.001 *
**Eat the same number of meals daily** Yes No	155 (37.5%) 257 (62.5%)	77 (49.7%) 131 (51.0%)	78 (50.3%) 126 (49.0%)	0.799
**Breakfast Time/day** Early breakfast (Before School) Late breakfast (in the school)	258 (63.0%) 153 (37.0%)	143 (55.4%) 64 (41.8%)	115 (44.6%) 89 (58.2%)	0.008 *
**Breakfast Regularly** Yes No	261 (63.4%) 151 (36.6%)	136 (52.1%) 72 (47.7%)	125 (47.9%) 79 (52.3%)	0.387
**Lunch Time/day** Early Lunch (before or at 1 P.M.). Late Lunch (after 1 P.M.).	75 (18.2%) 336 (81.6%)	41 (54.7%) 166 (49.4%)	34 (45.3%) 170 (50.6%)	0.410
**Lunch Regularly** Yes No	311 (75.5%) 101 (24.5%)	154 (49.5%) 54 (53.5%)	157 (50.5%) 47 (46.5%)	0.491
**Dinner Time/day** Early Dinner (before 7:30 P.M.) Late Dinner (after 7:30 P.M.)	19 (4.6) 391 (94.9)	7 (36.8%) 201 (51.4%)	12 (63.2%) 190 (48.6%)	0.215
**Dinner Regularly** Yes No	296 (71.7%) 116 (28.3%)	146 (49.3%) 62 (53.4%)	150 (50.7%) 54 (46.6%)	0.451
**Fasting hours/night**	10:54 ± 2:26	10:41 ± 2:22	11:07 ± 2:28	0.067
**Time between last meal and bedtime hours/night**	1:50 ± 1:10	1:56 ± 1:16	1:43 ± 1:02	0.063
**Most meals are important for the child** Breakfast Lunch Dinner	176 (42.6%) 182 (44.3%) 54 (13.1%)	92 (52.3%) 89 (48.9%) 27 (50.0%)	84 (47.7%) 93 (51.1%) 27 (50.0%)	0.814
**Eat Snacks Between Meals** Yes No	366 (88.9%) 46 (11.1%)	188 (51.4%) 20 (43.5%)	178 (48.6%) 26 (56.5%)	0.313
**Total daily energy intake (kcal/day)**	1550.15 ± 393.37	1608.56 ± 392.67	1490.60 ± 386.02	0.002 *
**Morning energy intake—before 6 P.M. (kcal)**	1006.66 ± 296.12	1040.97 ± 304.60	971.51 ± 283.63	0.017 *
**Evening energy intake—after 6 P.M. (kcal)**	547.48 ± 210.56	567.59 ± 205.21	526.88 ± 214.46	0.050 *
**Total fiber (g/day)**	10.46 ± 4.40	10.68 ± 4.64	10.25 ± 4.15	0.325
**Serving vegetables eaten/day**	0.26 ± 0.27	0.24 ± 0.25	0.29 ± 0.28	0.073
**Vegetables consumed/cup/day**	0.13 ± 0.13	0.12 ± 0.12	0.14 ± 0.14	0.078
**Serving fruit eaten/day**	0.31 ± 0.35	0.30 ± 0.34	0.32 ± 0.36	0.608
**Fruit consumed/cup/day**	0.15 ± 0.18	0.15 ± 0.17	0.16 ± 0.18	0.602

Data are presented as means ± SD or frequencies (%). Categorical variables were tested using the Chi-square test and continuous variables were tested using the independent *t*-test. * *p* < 0.05 was considered statistically significant.

**Table 5:** Physical fitness characteristics of the study sample according to BMI categories.

Variables	ALL (*n* = 412)	Non-Obese (*n* = 208)	Obese (*n* = 204)	*p*-Value
**20 mSRT**				
Total Laps	10.39 ± 9.15	10.40 ± 6.70	10.39 ± 11.12	0.990
VO_2_ MAX = mL/kg/min	32.90 ± 4.12	35.41 ± 2.81	30.50 ± 3.75	<0.001 *
**HGS (kg)**	16.09 ± 4.47	15.07 ± 4.09	17.13 ± 4.61	<0.001 *
**HGS/BMI**	0.76 ± 0.21	0.84 ± 0.21	0.68 ± 0.18	<0.001 *
**SLJ (cm)**	132.48 ± 31.11	132.53 ± 31.24	132.44 ± 31.05	0.976

Data are presented as means ± SD or frequencies (%). Categorical variables were tested using the Chi-square test and continuous variables were tested using the independent *t*-test. * *p* < 0.05 was considered statistically significant; 20 mSRT: 20 m shuttle run test; HGS: handgrip strength; HGS/BMI: relative handgrip strength/body mass index; SLJ: standing long jump.

**Table 6:** Fasting blood glucose and lipid profile of the study participants according to BMI categories.

Variable	ALL (*n* = 175)	Non-Obese (*n* = 81)	Obese (*n* = 94)	*p*-Value
**FBG (mg/dL)**	101.48 ± 14.82	102.78 ± 15.16	100.36 ± 14.52	0.450
**TC (mg/dL)**	121.27 ± 19.99	122.32 ± 20.75	120.37 ± 19.37	0.526
**HDL-C (mg/dL)**	51.62 ± 10.34	56.16 ± 11.34	47.70 ± 7.49	<0.001 *
**LDL-C (mg/dL)**	49.56 ± 19.12	46.19 ± 19.21	52.47 ± 18.65	0.408
**TG (mg/dL)**	102.77 ± 44.95	103.42 ± 46.64	102.20 ± 43.69	0.443

Data are presented as means ± SD. Continuous variables were tested using the independent *t*-test. * *p* < 0.05 was considered statistically significant. FBG: Fasting Blood Glucose; TC: Total Cholesterol; HDL-C: High-Density Lipoprotein; LDL-C: Low-Density Lipoprotein; TG: Triglyceride.

**Table 7:** Results of binary logistic regression analysis of chrononutrition, physical fitness, and biochemical variables associated with obesity status.

Variable	Crude Model			Adjusted Model		
	OR	95% CI	*p*-Value	aOR	95% CI	*p*-Value
**Chrononutrition behaviors**						
**Number of meals/days**	0.67	0.46–0.98	0.038 *	0.68	0.46–0.98	0.039 *
**Eat the same number of meals daily (ref: Yes)** No	1.06	0.68–1.64	0.805	1.04	0.67–1.61	0.872
**Breakfast Time (ref: Early breakfast: before School)** Late breakfast (in the school)	1.51	0.96–2.39	0.076	1.53	0.97–2.42	0.069
**Breakfast regularly (ref: Yes)** No	1.38	0.87–2.18	0.170	1.38	0.87–2.18	0.172
**Lunch Time/day (ref: Early Lunch: before 1 P.M.)** Late Lunch (after 1 P.M.)	1.05	0.61–1.82	0.860	1.06	0.61–1.84	0.845
**Lunch regularly (ref: Yes)** No	0.89	0.53–1.5	0.669	0.88	0.52–1.48	0.632
**Dinner Time/day (ref: Early Dinner: before 7:30 P.M.)** Late Dinner (after 7:30 P.M.)	0.71	0.26–1.97	0.510	0.73	0.26–2.02	0.540
**Dinner regularly (ref: Yes)** No	0.78	0.48–1.27	0.320	0.76	0.46–1.25	0.283
**Fasting Hours/night**	1.04	0.95–1.15	0.382	1.05	0.95–1.16	0.320
**Time between last meal and bedtime hours/night**	0.79	0.65–0.96	0.015 *	0.78	0.64–0.95	0.013 *
**Most important meal for the child (ref. breakfast)** Lunch Dinner	0.92 1.06	0.58–1.44 0.54–2.08	0.705 0.876	0.92 1.04	0.58–1.44 0.53–2.05	0.698 0.906
**Eat snacks between meals (ref: No)** Yes	0.98	0.5–1.89	0.941	0.96	0.5–1.87	0.908
**Evening energy intake—after 6 P.M. (% of total energy)**	1.00	0.98–1.02	0.931	1.00	0.98–1.02	0.981
**Physical fitness**						
**20 m shuttle run (laps)**	1.00	0.98–1.03	0.786	1.00	0.98–1.03	0.760
**Relative handgrip strength/BMI**	0.01	0.004–0.044	<0.001 *	0.01	0.002–0.030	<0.001 *
**Standing long jump (cm)**	1.00	1–1	0.628	1	1–1	0.904
**Biochemical** **markers**						
**Fasting blood glucose (mg/dL)**	0.98	0.96–1.01	0.130	0.98	0.96–1.01	0.129
**TC (mg/dL)**	1.00	0.96–1.04	0.913	1.00	0.96–1.04	0.913
**HDL (mg/dL)**	0.91	0.87–0.96	0.001 *	0.91	0.87–0.96	0.000 *
**LDL (mg/dL**	1.02	0.98–1.06	0.364	1.02	0.98–1.07	0.328
**Triglycerides** **(mg/dL)**	1	0.99–1.01	0.997	1.00	0.99–1.01	0.880

Note: OR = Odds Ratio; CI = Confidence Interval; aOR = Adjusted Odds Ratio. Adjusted models were controlled for age in all variables. For dietary intake variables derived from food records, additional adjustment for total daily energy intake was applied. The outcome variable was obesity status (obese vs. non-obese). Reference category: non-obese. * *p* < 0.05 indicates statistical significance.

## Data Availability

The data presented in this study are available from the corresponding author upon reasonable request due to ethical considerations and the protection of participants’ privacy.
